# Lemborexant‐induced interstitial lung disease: A case report

**DOI:** 10.1002/rcr2.1334

**Published:** 2024-04-25

**Authors:** Satoshi Nakahara, Yumiko Ishii, Reika Egashira, Kazuya Tsubouchi, Mikihiro Kohno, Tomoyoshi Takenaka, Kentaro Tanaka, Isamu Okamoto

**Affiliations:** ^1^ Department of Respiratory Medicine, Graduate School of Medical Sciences Kyushu University Fukuoka Japan; ^2^ Department of Surgery and Science, Graduate School of Medical Sciences Kyushu University Fukuoka Japan

**Keywords:** drug‐induced interstitial lung disease, lemborexant, orexin receptor antagonists, type IV hypersensitivity reactions

## Abstract

We report the first case of drug‐induced interstitial lung disease attributed to lemborexant. A 66‐year‐old man reported to our hospital with the acute onset of cough and breathlessness with ground‐glass opacity on radiological examination. Symptoms were identified after taking lemborexant for 2 consecutive days. The patient had undergone lemborexant treatment 2 years prior and had exhibited no symptoms at that time. The drug‐induced lymphocyte stimulation test for lemborexant was positive. He showed rapid improvement upon treatment with steroid. With the rise in prescriptions of lemborexant for insomnia, lemborexant should be considered as a possible cause of drug‐induced interstitial lung disease.

## Graphical abstract

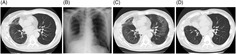

## INTRODUCTION

Drug‐induced interstitial lung disease (DIILD) is an adverse drug reaction commonly due to disease‐modifying antirheumatic drugs (DMARDs), antimicrobial, and antineoplastic drugs including immune checkpoint inhibitors. Lemborexant, an orexin receptor antagonist (ORA), has been used for the treatment of insomnia following its approval by the Food and Drug Administration (FDA) in 2019 and lemborexant‐induced ILD has not been reported previously. We report a case of DIILD attributed to lemborexant.

## CASE REPORT

A 66‐year‐old man underwent right lung lobectomy for lung cancer without adjuvant therapy. He developed right lung pneumonia 1 month after the lobectomy. One and half months after the recovery from pneumonia, he developed acute onset of cough and breathlessness after taking lemborexant for two consecutive days for insomnia, which he had previously had three or four times without side effects 2 years ago. He presented to the hospital on the next day of the onset. He had not started any other medication except for lemborexant. The oxygen saturation was 92% on supplemental oxygen therapy of 3 L/min at rest.

**FIGURE 1:**
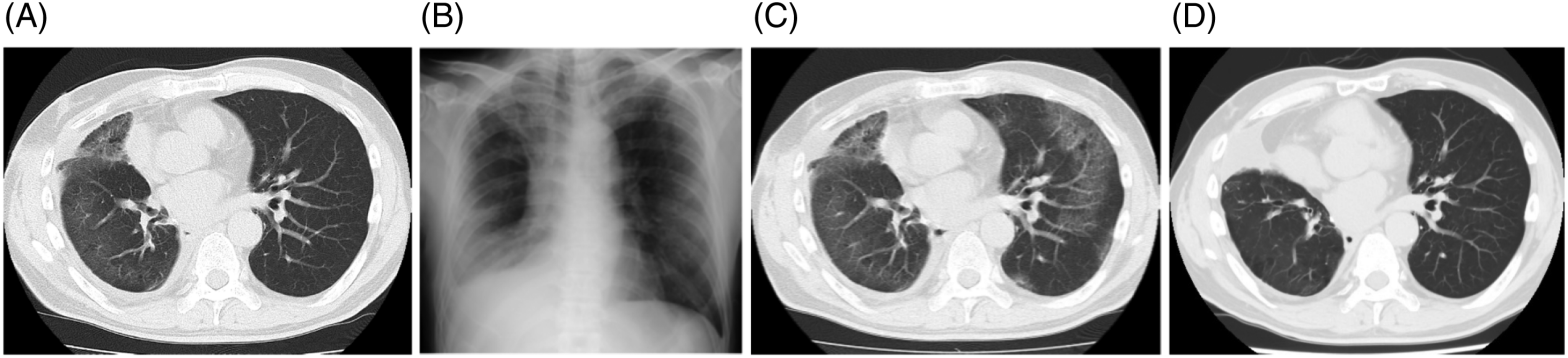
Images of chest x‐ray and computed tomography (CT). (A): day 7, (B) and (C): day 0, and (D): 9 months after the onset.

**FIGURE 2:**
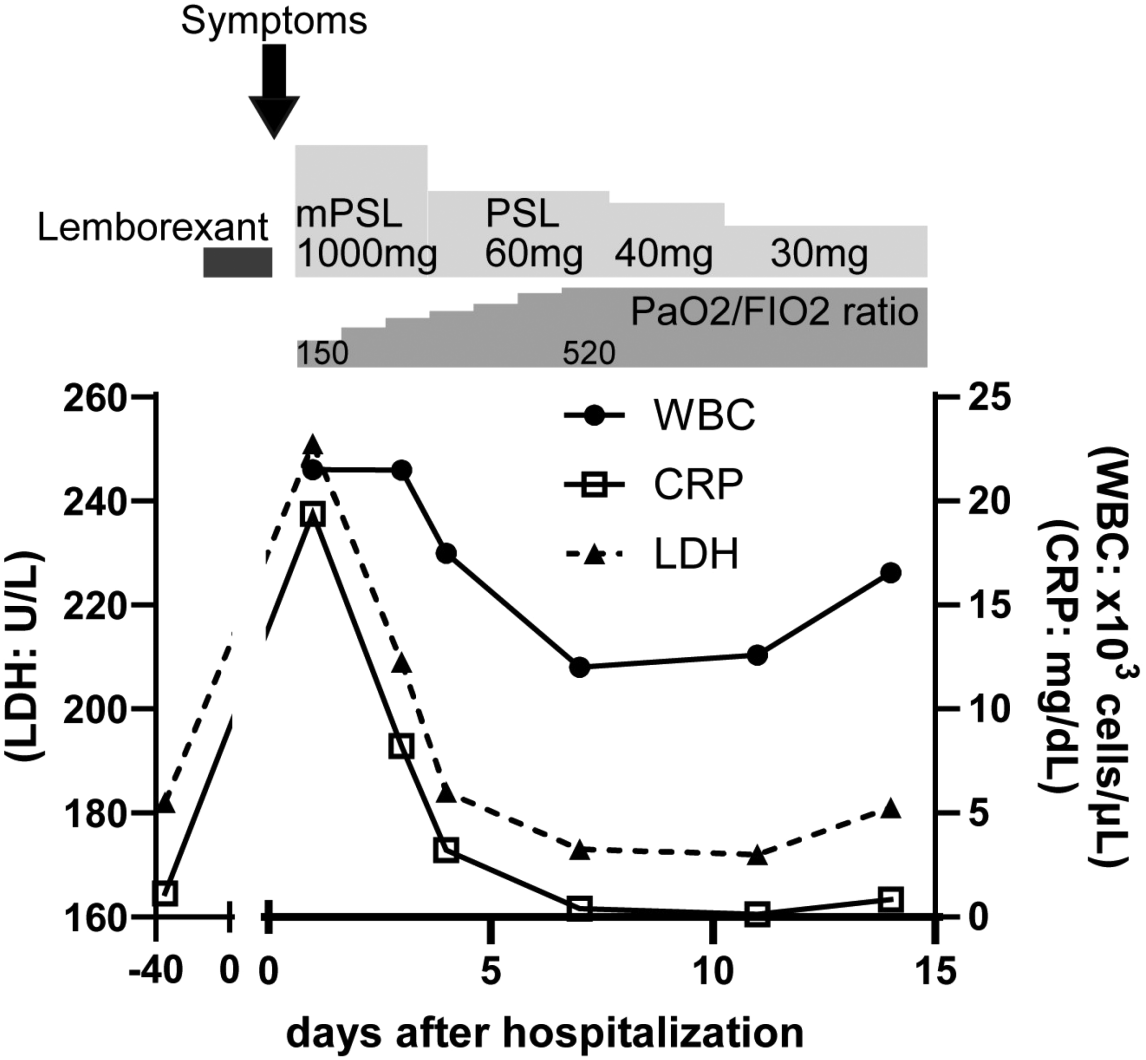
Clinical course of the patient. The patient had cough and breathlessness with GGO and the elevated CRP after taking lemborexant 2 consecutive days. Following steroid pulse therapy and discontinuation of lemborexant, the PaO_2_/FIO_2_ ratio, LDH, and CRP levels improved rapidly.

Chest x‐ray and computed tomography (CT) scan showed diffuse ground‐glass opacity (GGO) with fine crackles in the left lung (Figure 1). CT scan at 7 days before the onset revealed the normal left lung. The white blood cell counts (11,210 cells/μL), eosinophil counts (336 cells/μL), and C‐reactive protein (CRP) levels (19.39 mg/dL) were elevated; however, the serum Krebs von den Lungen‐6 (KL‐6), surfactant protein‐D (SP‐D), antinuclear antibodies, and β‐D‐glucan levels were within the normal range. Urinary pneumococcal antigen and urinary Legionella antigen were negative. Sputum was negative for common bacteria and acid‐fast bacilli. FilmArray Panel Tests (BIOMERIEUX Japan, Cat: 423742) for viruses and bacteria were negative. The drug‐induced lymphocyte stimulation test (DLST) was positive for lemborexant. There were no findings suggestive of viral, bacterial, and/or fungal infections, and the patient was diagnosed with DIILD attributed to lemborexant. As the patient's respiratory condition rapidly deteriorated following hospitalization, bronchoscopy with bronchoalveolar lavage (BAL) was not performed. Following steroid pulse therapy (1000 mg/day for 3 days) and discontinuation of lemborexant, prednisolone was started at a dose of 60 mg/day (equivalent to 1 mg/kg/day). The PaO_2_/FIO_2_ ratio, GGO, and CRP levels improved rapidly, and the prednisolone dose was subsequently tapered over 9 months (Figure 2). He was never re‐exposed to lemborexant subsequently.

## DISCUSSION

Insomnia is a common condition characterized by a predominant complaint of dissatisfaction with the sleep quantity or quality. The coronavirus disease pandemic has been associated with an increased prevalence of insomnia (19% in 2020).^1^ ORAs exhibit fewer side effects such as carry‐over effect and cognitive impairment than other commonly prescribed drugs for insomnia, such as benzodiazepines. Additionally, network meta‐analysis revealed that lemborexant was the best profile from the aspects of efficacy, acceptability, tolerability, and safety.^2^ Therefore, the proportions of patients with insomnia receiving ORA has been increasing from 0% in 2013 to 20% in 2019.^3^

DIILD has a prevalence of 3–5% worldwide. The risk factors for the development of DIILD are (1) age (>60 years); (2) sex (males > females); (3) pre‐existing lung diseases, including interstitial lung disease, lung cancer, and chronic obstructive pulmonary disease (COPD); and (4) smoking and other factors.^4^ The patient was a 66‐year‐old man and exhibited complications of lung cancer and COPD. Additionally, the patient had postoperative pneumonia. These factors could have contributed to the increased susceptibility to DIILD.

An intact left lobe was confirmed by a chest CT scan 7 days before hospitalization. The history and timing of drug exposure, prompt improvement after drug discontinuation, glucocorticoid administration, and DLST results support the diagnosis of DIILD attributed to lemborexant. KL‐6 is secreted by the bronchial epithelial cells and damaged type II alveolar pneumocytes. A normal KL‐6 level does not reduce the possibility of DIILD as increased KL‐6 levels were not reported in 47% of patients with DIILD in a previous study.^5^

The primary pathogenic mechanisms underlying DIILD are allergic and toxic reactions. Toxic reactions are often evoked by anticancer drugs. Non‐toxic drugs, such as antibiotics and amiodarone, are involved in DIILD caused by allergic reactions, especially type IV hypersensitivity reactions. Sensitization of the T cells prior to their activation owing to contact with the allergens is necessary in Type IV hypersensitivity reactions. Hypersensitivity reactions usually occur more than 12–72 h following exposure to allergens; however, the duration between allergen exposure and onset is approximately 1 month in DIILD. In this case, the patient had taken lemborexant 2 years before hospitalization and had not shown any symptoms. The T cells could have been sensitized to lemborexant at the first episode of oral administration. In addition, the DLST results are positive more often in type IV hypersensitivity reactions than in other types of allergic reactions. The short duration from the time of administration of the second course of lemborexant to the onset of symptoms and the presence of a positive DLST result suggested a type IV allergy.

There had been no reports of the DIILD attributed to lemborexant previously. This is the first case report of lemborexant‐induced interstitial lung disease and the predictive mechanism was type IV hypersensitivity reaction. Thus, based on the findings of the present case report, lemborexants should be considered as a possible cause of drug‐induced interstitial lung disease.

## AUTHOR CONTRIBUTIONS

S.N. wrote the original draft preparation. Y.I. and I.O. reviewed and supervised. All authors contributed to the editing of the manuscript and approved the final version of the manuscript.

## CONFLICT OF INTEREST STATEMENT

Non declared.

## ETHICS STATEMENT

The authors declare that appropriate written informed consent was obtained for the publication of this manuscript and accompanying images.

## Data Availability

The data that support the findings of this study are available on request from the corresponding author. The data are not publicly available due to privacy or ethical restrictions.
